# *Salmonella enterica*: Survival, Colonization, and Virulence Differences among Serovars

**DOI:** 10.1155/2015/520179

**Published:** 2015-01-13

**Authors:** A. Andino, I. Hanning

**Affiliations:** Department of Food Science and Technology, University of Tennessee, 2605 River Drive, Knoxville, TN 37996, USA

## Abstract

Data indicate that prevalence of specific serovars of *Salmonella enterica* in human foodborne illness is not correlated with their prevalence in feed. Given that feed is a suboptimal environment for *S. enterica*, it appears that survival in poultry feed may be an independent factor unrelated to virulence of specific serovars of *Salmonella*. Additionally, *S. enterica* serovars appear to have different host specificity and the ability to cause disease in those hosts is also serovar dependent. These differences among the serovars may be related to gene presence or absence and expression levels of those genes. With a better understanding of serovar specificity, mitigation methods can be implemented to control *Salmonella* at preharvest and postharvest levels.

## 1. Introduction

Salmonellae are facultative anaerobic Gram-negative rod-shaped bacteria generally 2–5 microns long by 0.5–1.5 microns wide and motile by peritrichous flagella. Genome sizes of* Salmonella* vary among serovars (Table 1) with ranges from 4460 to 4857 kb. Salmonellae belong to the family Enterobacteriaceae and are a medically important pathogen for both humans and animals. Salmonellae form a complex group of bacteria consisting of two species and six subspecies and include more than 2,579 serovars [1, 2]. Two species are currently recognized in the genus* Salmonella*,* S. enterica* and* S. bongori* [3].* S. enterica* can be subdivided into the subspecies* enterica*,* salamae*,* arizonae*,* diarizonae*,* houtenae*, and* indica* based on biochemical and genomic modifications [4]. The majority of* Salmonellae* are lactose fermenters, hydrogen sulfite producers, oxidase negative, and catalase positive. Other biochemical properties that allow identification of* Salmonella* include the ability to grow on citrate as a sole carbon source, decarboxylate lysine, and hydrolyze urea [5, 6].

The main niche of* Salmonella* serovars is the intestinal tract of humans and farm animals. It can also be present in the intestinal tract of wild birds, reptiles, and occasionally insects. Feedstuff, soil, bedding, litter, and fecal matter are commonly identified as sources of* Salmonella* contamination in farms [7–10]. As* Salmonella* colonizes the gastrointestinal tract, the organisms are excreted in feces from which they may be transmitted by insects and other animals to a large number of places and are generally found in polluted water. Salmonellae do not originate in water; therefore their presence denotes fecal contamination [6]. Humans and animals that consume polluted water may shed the bacteria through fecal matter continuing of the cycle of contamination.

Like many other infectious diseases, the course and outcome of the infection depend on variable factors including the dose of inoculation and the immune status of the host [11]. In the US,* Salmonella* is the leading foodborne pathogen, causing the largest number of deaths and has the highest cost burden [12]. The annual costs associated with salmonellosis for 2010 were estimated at $2.71 billion for 1.4 million cases [13]. The highest numbers of* Salmonella* outbreaks from the past decade are related to land animals, with poultry as a main reservoir (Table 2). More than 70% of human salmonellosis in the US has been attributed to the consumption of contaminated chicken, turkey, or eggs [14]. From 1998 to 2008, approximately 145* Salmonella* outbreaks have been associated with poultry while 117 outbreaks were associated with eggs, causing illnesses in 2580 and 2,938 people, respectively [14].

However, a considerable number of outbreaks have been related to crops (Table 3). From 1998 to 2008 fruits and nuts were the highest cause of* Salmonella* outbreaks in plant products, followed by vine stalk vegetables and sprouts. Sanderson and Demerec [11] reported that* Salmonella* appears eight times between the top 20 ranked pathogen-food combinations and is most notably associated with poultry, produce, and eggs. It is not always easy to identify specific serovars in an outbreak; in many cases* Salmonella* cannot be linked to a specific food component due to complex food preparations using a variety of ingredients.

In the US, data from foodborne outbreaks related to human illness collected from 2007 to 2011 reported that 89% were confirmed serotypes [14]. Serovar Enteritidis was the most frequently isolated followed by Typhimurium, Newport, Heidelberg, and Montevideo (Table 4). The food vehicles associated with this serovars include a wide variety of products including eggs, chicken, pork, leafy greens, peanut butter, turkey, dairy products, and vegetables (Table 4).

Salmonellae can enter and survive in the farm environment for long periods of time. Prevalence of* Salmonella* in farm environments ranges from 10 to 26% according to a recent study [9]. Presence of* Salmonella* in feed and feed ingredients is well documented [9, 31–33]. However, very low levels of* Salmonella* have been obtained from drinking water samples from broiler farms. Conversely, recovery of* Salmonella* was easily accomplished in samples from standing water where the bacteria can persist in biofilms [31, 32, 34]. Variety and prevalence of* Salmonella* serovars differ among studies in different regions and types of farms. Yet, there is some consistency in recovery rates of specific serovars: Heidelberg, Kentucky, Enteritidis, Typhimurium, Montevideo, Seftenberg, and Thompson as these are the highest recovered serotypes [32, 34, 35]. In a one year experiment in an integrated operation, Bailey et al. [32] found that hatchery transport pads, flies, drag swabs, and boot swabs exhibited the highest prevalence of* Salmonella*. The most frequently identified serotypes from those farm samples were Seftenberg, Thompson, and Montevideo. According to reports from the monitoring system by the USDA through the Food and Safety Inspection Service (FSIS), from 2000 to 2009 serotypes Kentucky, Enteritidis, Heidelberg, Typhimurium, and antigenic formula I 4, 5,12:i:- were commonly isolated from broilers (Table 5) and ground chicken (Table 6).

Shell eggs are a major vehicle for* S.* Enteritidis in humans. By 1994* S.* Enteritidis became the most frequent serovar reported in the US causing human salmonellosis. From 1985 to 2003 in 75% of* S.* Enteritidis outbreak cases, eggs were confirmed as the primary ingredient or food vehicle of contamination [14]. A major outbreak occurred in 1994 where tanker trailers that previously carried* S.* Enteritidis contaminated liquid eggs cause the cross contamination of ice-cream prepared at the same facility [37]. Serovar Enteritidis is known to be very well adapted to the hen house environment, the bird, and the egg. Most commonly, hens are infected with* S.* Enteritidis by vertical transmission and through transovarian infection eggs may become contaminated [38].* S.* Typhimurium and other serovars usually contaminate eggs externally by penetrating the egg shell [39]. Surveys conducted in US report* Salmonella* contamination in table eggs by other serovars including Heidelberg and Montevideo [39, 40]. Enhanced biosecurity practices, postharvest intervention methods (sanitizing and decontamination), and egg pasteurization can reduce the risk factors for* Salmonella* infection in laying hen operations [41].

## 2. *Salmonella* Serovar Host Specificity and Disease

### 2.1. Diseases in Chickens

Birds infected with most* Salmonella* serovars do not show clinical signs of the disease, making it difficult to diagnose at the farm. However,* S.* Pullorum and* S.* Gallinarum do cause disease in poultry but rarely cause illness in humans. These* Salmonella* serovars are nonmotile and host-specific and cause Pullorum disease (PD) and fowl typhoid (FT), respectively [42].

Pullorum disease was first described as “fatal septicemia” or “white diarrhea” [42]. Clinical signs are predominantly observed in young chickens, showing lack of appetite, depression, respiratory distress, caseous core diarrhea, and early death a few days after hatching. In laying hens symptoms include reduced egg production, fertility, and hatchability [43–45].* Salmonella* Pullorum may cause severe systemic lesions including peritonitis and liver and spleen enlargement, and organs may be streaked with hemorrhages; animals can also develop white focal necrosis in the case of young birds and abnormal color and shape in ovaries in older birds. Pullorum disease mortality rate is variable but maybe as high as 100% in critical cases.

Fowl typhoid disease is caused by* S.* Gallinarum and affects chickens, turkeys, guinea fowl, and birds of all ages and breeds [46]. The first described outbreak was characterized by high mortality and signs of the disease that began with yellow-to-green diarrhea with the birds dying few days after infection [42]. Unlike* S.* Pullorum,* S.* Gallinarum is more frequently seen in older birds than young birds. One of the first signs of this disease is an increase in mortality rate, followed by a decline in feed consumption and therefore a drop in egg production and weight gain [44]. Histological examination reveals fatty degeneration of the liver, occasionally accompanied by areas of necrosis, disintegration of muscle fibers, and congestion and perivascular infiltration of mononuclear cells in the kidneys [47].

*Salmonella* Pullorum and* S.* Gallinarum have been eradicated in developed regions including the US, Canada, and Western Europe but are still problems in other parts of the world. Control programs that incorporated good hygiene management, biosecurity enforcement, serological tests, and slaughter policies helped with the eradication of these pathogens. In 1935, the US Federal Government executed the National Poultry Improvement Plan (NPIP) in order to reduce the mortality of chickens from Pullorum and Gallinarum disease. In the 1950s, poultry breeders and hatchers in US implemented tests (blood analysis, tube agglutination, and rapid serum test) for* S.* Pullorum and* S.* Gallinarum on a regular basis while uniform national management standards were adopted. Furthermore, in the 1950s vaccination was implemented to control Pullorum disease and fowl typhoid. Two decades later both diseases were eradicated and by 1975 there was no evidence of infection in commercial poultry [43, 48, 49].

It has been suggested that clearing poultry flocks of* S.* Gallinarum and* S.* Pullorum opened a favorable niche for* S.* Enteritidis [50–52]. The use of mathematical models with data from Europe and US suggests that* S.* Gallinarum excluded* S.* Enteritidis from poultry [53]. Coincidently,* S.* Enteritidis detection was on the rise after eradication of* S.* Gallinarum and* S.* Pullorum, and by the 1990s it was the most frequently reported serovars in the US. Unlike avian* Salmonella* pathogens, serovar Enteritidis has rodents as reservoirs, making it more difficult to control on the farms.* S.* Enteritidis and* S.* Gallinarum are antigenic similar, both belonging to serogroup D1 possessing a similar lipopolysaccharide structure and O9 antigens. When commercial flocks were cleared from* S.* Gallinarum, serovar Enteritidis was able to colonize chickens without noticeable signs of disease. It is believed that seropositive* S.* Pullorum had an enhanced immunity dominant O9 antigen that protected against* S.* Enteritidis infection [50].

### 2.2. Diseases in Humans

#### 2.2.1. Typhoid and Paratyphoid Fevers

Clinically, salmonellosis may be manifested as gastroenteritis, septicemia, or enteric fever. Enteric fevers are caused by the human-specific pathogens* S. enterica* serovars Typhi and Paratyphi. Infection severity may vary by the resistance of each individual and the immune system as well as the virulence of the* Salmonella* isolate.

*Salmonella* Typhi is a motile, nonlactose fermenting bacillus that causes most endemic and epidemic cases of typhoid fever globally [54, 55]. Enteric fevers cause 200,000 deaths and 22 million illnesses per year, with the highest incidence happening in Southeast and Central Asia where it is endemic [56]. Doses from 10^3^ to 10^9^ CFU of* Salmonella* Typhi are known to cause enteric fever [57].

#### 2.2.2. Nontyphoidal Salmonellosis

Like enteric fevers, nontyphoidal salmonellosis (NTS) is spread via the fecal-oral route, but estimated cases of NTS worldwide greatly surpass those for enteric fevers. Unlike Typhi and Paratyphi, nontyphoidal Salmonellae are not human-restricted. Many serovars closely related to foodborne outbreaks include* S.* Typhimurium,* S.* Enteritidis,* S.* Newport, and* S.* Heidelberg and have reservoirs in farm animals [9, 58]. Among other foodborne pathogens, NTS is the leading cause of death and hospitalizations [59]. In NTS, cases are characterized by gastroenteritis or bacteraemia; symptoms may involve nausea, vomiting, and diarrhea and are typically self-limiting lasting approximately 7 days.* Salmonella* can also induce chronic conditions including aseptic reactive arthritis and Reiter's syndrome.

### 2.3. Differences among Serovars with respect to Disease Severity

Different* Salmonella* serovars may demonstrate unique reservoirs and pathogeneses. It is still poorly understood why a few* Salmonella* serovars are responsible for a majority of human diseases, but nearly all of them belong to subspecies* enterica*. In a 1995 global survey, serotypes Enteritidis and Typhimurium were the most prevalent serovars of all isolates [60]. The biggest difference among severity and treatment methods is between enteric fever salmonellae and nontyphoid salmonellae (Table 7). It is suggested that a combination of factors specific to each serovar including the presence of plasmid virulence genes (*spv*), surface cell structure, flagellin, and pathogenity islands (SPIs) is involved in severity of salmonellosis. It has been demonstrated that* S.* Seftenberg and* S.* Litchfield have large deletions in invasion related genes, which might have been the result of a selective advantage in the intestinal environment [61]. Jones et al. [62] analyzed data from more than 50 salmonellosis cases from 1996 to 2006 assessing differences among serovars in terms of severity. From these data, most illnesses were related to serovars Typhimurium, Enteritidis, and Newport, while fatality rates reported were in most cases related to serovars Dublin, Muenster, and Choleraesuis.

### 2.4. Differences among Serovars with respect to Antibiotic Resistance

Resistant* Salmonella* strains are commonly found in food animal sources [63, 64]. Mismanagement of antimicrobial agents for treatment in humans and animals and the use of growth promoters in livestock have promoted antimicrobial resistance in Salmonellae [64, 65]. The occurrence of* Salmonella* serovars resistant to quinolones, fluoroquinones, and third generation cephalosporins which are medically significant treatments has increased [66–68]. According to a NARMS report in 2010, the serovars with greater resistance to antimicrobials are Typhimurium specific to ampicillin, chloramphenicol, streptomycin, sulfamethoxazole/sulfisoxazole, and tetracycline (ACSSuT), as well as Enteritidis with resistance to naldixic acid. Serovars Newport, Heidelberg, Dublin, and I4, [5], 12:i:- were also shown to be resistant to various antimicrobial groups (Table 8). In terms of multidrug resistance (more than 5 antimicrobials) the most prevalent serovars of epidemiological importance are Typhimurium, Heidelberg, Dublin, Paratyphi B, and I4, [5], 12:i:- (Table 9). Although* S.* Enteritidis is highly prevalent in human infections; it has lower antimicrobial resistance compared to other serovars. Antimicrobial resistance in* Salmonella* can be associated with horizontal transference of antibiotic resistant genes characteristically found on mobile genetic elements among* Salmonella* strains and other Enterobacteria or by clonal spread of antimicrobial drug resistant serovars that are particularly effective in worldwide dissemination [69–72]. The mechanisms from which* Salmonella* develops resistance include production of enzymes that can degrade cell permeability to antibiotics, activation of antimicrobial efflux pumps, and production of *β*-lactamase to degrade the chemical structure of antimicrobial agents [73, 74].

Farm animals have been a common source of isolation for antimicrobial resistant* Salmonella* serovars [76–78]. A predominantly infectious* S.* Typhimurium DT104 emerged in the 1980s and has managed to spread worldwide. This serovar commonly carries chromosomally based resistance to five antimicrobials (ACSSuT) and it is believed that it was disseminated worldwide by human travel and then spread locally by the absence of effective antimicrobials [76, 79, 80].* Salmonella* Newport has been identified to harbor plasmids encoding ACSSuT and produces *β*-lactamase, which inactivates cephalosporins, providing resistance to ampicillin and chloramphenicol (AmpC). In human isolates from* S.* Heidelberg showing high invasive infections, large plasmids (IncA/C and IncI1) were found to carry multiple resistance genes [65, 81]. It is believed that horizontal transmission of virulence genes in multidrug resistant* Salmonella* strains can increase virulence and invasiveness and cause higher mortality rates compared to susceptible* Salmonella* [79, 81, 82].

## 3. Prevalence on the Farm

### 3.1. Cattle

Salmonellosis in cattle is caused by numerous serovars, with* S.* Typhimurium and* S.* Dublin being the most common [83].* Salmonella* Dublin serovar is commonly detected in calves and adult cattle. Most infections are introduced into* Salmonella* free herds by the purchase of infected animals that might have acquired infection on farm premises, in transit or on dealer's premises [84]. Another route of contamination can be waterborne infection. During the early stages of the acute enteric disease affected animals develop fever, dullness, loss of appetite, and depressed milk yield and adult pregnant animals may abort [83, 85]. Infection of* S.* Dublin in humans is commonly developed after contact with carrier animals but can also be transmitted through contaminated food and may cause gastroenteritis [86, 87].

In samples taken by FSIS/USDA from 2000 to 2009 from cows and bulls, the increasing prevalence of serovars Montevideo, Newport, Agona, Kentucky, and Mbandanka is notable over the last decade (Table 10). Furthermore, when steers and heifers were submitted to the same testing* S.* Dublin,* S.* Montevideo,* S.* Typhimirium,* S.* Anatum, and* S.* Newport were more prevalent than other serovars (Table 11). Beef products are among the top five products related to* Salmonella* foodborne outbreaks (Table 2). When ground beef was tested, a constant increase in* S.* Montevideo and* S.* Dublin isolates was detected from 2004 to 2009, followed by serovars Newport, Typhimurium, and Anatum (Table 12). A multistate sample collection from dairy cows reported that 7.3% of the samples were positive for* Salmonella* and the five most predominant serotypes were Meleagridis, Montevideo, Typhimurium, Kentucky, and Agona [88]. However, 83% of the isolates were susceptible to all the antimicrobial drugs tested.

### 3.2. Pigs

Pigs are an important reservoir of human nontyphoidal salmonellosis and the isolation of the organism from pork and pork products is very common. Porcine salmonellae consist of two groups separated by host range and clinical presentation. The first group consists of the host-adapted serovar* S.* Choleraesuis and tends to elicit systemic disease in the form of septicaemia with a high mortality rate in young pigs. The second group consists of all the other serovars, which have a broader host range and tend to produce momentary enteritis. Like other animal farms, the prevalence of* Salmonella* from swine varies depending on the region and type of farm surveyed. Prevalence of* Salmonella* in samples taken from swine farm environments ranges from 3 to 33% [9, 89, 90]. When fecal samples were taken from grower and finisher pigs, the prevalence among serovars was higher for* S.* Derby and* S.* Typhimurium followed by Agona and Anatum, which are among the serovars with highest incidence in human foodborne outbreaks [91]. Moreover, 79.6% isolates were resistant to at least one antibiotic [91]. Antimicrobial resistance has been more likely associated with* S.* Typhimurium and* S.* Derby and pigs can become asymptomatic carriers [92].

In the US, from 2000 to 2009 the most prevalent serovars isolated from market hogs were Derby, Typhimurium, Johannesburg, Infantis, and Anatum, two of which were also in the top five serotypes isolated from humans in the same period [36]. Other serovars commonly isolated from pigs in recent years include Heidelberg, Saintpaul, and Agona (Table 13). Since the early 1990s there has been a shift in the predominant serovar isolated from swine, where Choleraesuis has a higher incidence and replaced* S.* Typhimurium.

### 3.3. Poultry

Chicks may acquire* Salmonella* via vertical transmission from the parent, but horizontal transmission from environmental facilities, transportation, feed, and vectors including humans, rodents, and insects can be a significant problem [90, 93]. Among commercial layers, contaminated eggs will typically result from flock infections acquired via persistent environmental* Salmonella* and are associated with the serovar Enteritidis [94–96]. In studies conducted in poultry farms,* Salmonella* prevalence ranges between 5 and 100% among various environmental and fecal samples [9, 97–99]. It appears that* Salmonella* Enteritidis filled an ecological niche that was available after eradication of serovars Pullorum and Gallinarum.* S.* Enteritidis was the most prevalent serovar isolated from chickens during the 1990s but that has changed in the following decade. In recent years the serotypes commonly associated with chickens are Enteritidis, Kentucky, Heidelberg, Typhimurium, and I 4, [5], 12:i:- (Tables 5 and 6).

### 3.4. Food Products

*Salmonella* outbreaks linked to consumption of nonmeat foods have rapidly increased during the last decades. Recent data indicates that 13% of the* Salmonella* outbreaks in the US have been related to contaminated nonmeat foods [100, 101].* Salmonella* Saintpaul,* S.* Rubislaw, and* S.* Javiana spread by paprika and paprika-powdered potato chips caused outbreaks with more than 1000 infected people [102]. An increase of* S.* Oranienburg infections was registered in the early 2000s where multistate control studies revealed the consumption of chocolate as the apparent cause of infection [103]. Epidemiological and environmental investigations indicate that cross contamination in the manufacturing plants may be the cause of outbreaks associated with low moisture foods [104].* Salmonella* Typhimurium,* S.* Ofda,* S.* Tennessee, and* S.* Poona were isolated from sesame paste and sesame seed which were sold for raw consumption in cereals [105]. It is known that bacteria on plant surfaces may form large biofilms with other bacteria [106]. The persistence of these biofilms makes it difficult to clean and sanitize the crops. These factors are thought to contribute to outbreaks related to plant products including fruits, nuts, and vine stalk vegetables as common sources (Table 3). Outbreaks of salmonellosis associated with seafood that occurred in the US were from cross contamination during farming, processing, preparation, and transportation. From 1999 to 2011, serovars Newport, Typhimurium, Dublin, Montevideo, and Java were reported to have caused outbreaks associated with consumption of milk and cheese products in the US [104]. The reason some* Salmonella* serovars are more prevalent in specific food products is not completely understood. It is suggested that Salmonellae react in a serovar dependent manner to environmental stresses including differences in temperature, chemicals, and low-nutrient available conditions that can vary by food [107–109].

## 4. Survival and Stress

### 4.1. Temperature

*Salmonella* is considered to be mesophilic with some strains being able to survive at extremely low or high temperatures (2°C to 54°C). Sigma factors are proteins that compose fundamental subunits of prokaryotic RNA polymerase and provide a mechanism for cellular responses by redirecting transcription initiation [110]. Alternate sigma factors control the gene expression of bacteria by sensing the changes in the environment. The sigma factors can sense perturbation in the outer membrane and activate genes in response to heat stress in order to adapt to high temperatures. The mechanism used is by specific activation and transcription of* rpo*H genes under high temperature [111]. Transcription of* rpo*H genes in* S.* Enteritidis was at the highest level when cultured at 42°C. Additionally all virulence genes were upregulated in response to high temperature [112, 113].

Water activity (*a*_*w*_) in foods is defined as the ratio of the vapor pressure of water in a food matrix compared to that of pure water at the same temperature. Extended time and temperature are required to kill 90% of* Salmonella* populations (*D*-value) in low *a*_*w*_ foods and may reflect the low efficiency of thermal inactivation in dry foods involved in* Salmonella* related outbreaks including flour, nuts, butter, dry milk, and chocolate [104, 114]. The surrounding moisture and the conformation of the food matrix can influence the thermotolerance of* Salmonella* by increasing the temperature required to inactivate the organism. Under low *a*_*w*_ conditions in high carbohydrate or high fat products, the heat resistance of* S.* Seftenberg strain 775W was greater than* S.* Typhimurium [115–118]. It is widely known that* S.* Seftenberg strain 775W has high resistance to heat, with a thermotolerance approximately 30 times more than* S.* Typhimurium. The thermotolerance of* Salmonella* in poultry products including liquid egg yolks and chicken meat highlights the distinctiveness of* S.* Seftenberg to survive high cooking temperatures. Other strains of* S.* Seftenberg and* S.* Bedford have shown similar inactivation temperatures to strain 775W.* Salmonella* Senftenberg and* S.* Typhimurium exhibited higher resistance to heat in chicken litter among other* Salmonella* serovars [119–121]. Heat stress encountered during feed processing increased the thermotolerance of* S.* Enteritidis strains and may induce expression of virulence gene* hil*A in* S.* Enteritidis,* S.* Typhimurium, and* S.* Seftenberg [122, 123]. It is believed that heat resistance confers a preadaptation to temperatures and it is influenced by the strain tested and culture conditions [124, 125].

*Salmonella* uses cold shock proteins (CSP) as a response for quick adaptation to temperature downshifts in the environment. The CSPs are created during the acclimation phase from 30°C to 10°C. During the downshift CSPs are synthesized for the cell to later resume growth [126–128]. Many studies have been conducted on the ability of salmonellae to increase its survival rate by expressing a CSP when treated at low temperature (5°C to 10°C) prior to freezing.* S.* Enteritidis was able to survive in chicken parts at 2°C, and in shell eggs at 4°C, while* S.* Typhimurium survives in minced chicken at 2°C;* S.* Panama has also shown an elevated propensity to survive in agar at 4°C and* S.* Typhimurium and* S.* Tennessee had the ability to survive in estuarine environments below 10°C [129].

### 4.2. Chemicals

There are a wide variety of potential chemical stresses, including pH, oxidation, membrane disruption, and denaturation of critical macromolecules or metabolic poisons that can affect pathogenic bacteria [130, 131]. Chlorine, commonly used to disinfect water, can be antimicrobial to* Salmonella*. Salmonellae are capable of producing biofilms providing the organism with an exopolysaccharide matrix that inhibits chemical attack against chlorine [132–134]. Chlorine in recommended doses (2–5 ppm of available chlorine) is able to prevent bacterial biofilm formation in poultry drinking systems and reduce the incidence of* Salmonella* in the crop and ceca of broilers [135, 136]. However, chlorination by itself is not enough to reduce* Salmonella* incidence and infection in birds [137].

Decontamination of broiler carcasses occurs during immersion in the chilling tank and the bacterial load in each carcass is expected to be lower than an initial count. The use of chlorine at range of 20–50 ppm in the chilling tank is enough to remove* Salmonella* biofilm on stainless steel. Chlorine is also used as a sanitizing method in poultry processing plants along with organic acids, inorganic phosphates, and other organic preservatives. Treatments for decontamination of carcasses were performed on different strains of* Salmonella* in the presence of acidified sodium chlorite varied widely with serotype; the highest resistance levels were shown by serotypes Typhimurium, Newport, and Derby [138]. Among organic acids the use of acetic and propionic acid has shown inhibitory effects against* Salmonella* [139, 140]. Equipment sanitization is also important, and previous studies have shown the importance of combining sanitizing agents, including detergents and acids. Treatments with sanitizers and detergent successfully inactivated* S.* Enteritidis cells compared with a 50% inactivation by using sanitizers only [141]. In general, chlorate preparations act as selective toxic agents to enteric pathogens by disrupting cell membrane causing the leakage of intracellular components in bacterium.

### 4.3. pH

In the case of organic acids their bactericidal activity is related to pH, affecting creation of undissociated acids that will acidify the cytoplasm and disrupt key biochemical processes. In chickens,* Salmonella* first reaches the crop with a pH range of 4 to 5, as a result of bacterial lactic acid fermentation. If adaptation to that pH occurs,* Salmonella* can survive and adapt to a more acidic pH and therefore oppose antibacterial effects of the stomach [142].

Many virulence factors in bacteria, including* Salmonella*, are regulated via the PhoP/PhoQ system. PhoP genes act on the bacterial cell envelope by increasing the resistance to low pH and enhancing survival within the macrophage [143].* Salmonella* responds to acidic environmental challenges of pH 5.5 to 6.0 (preshock) followed by exposure of the adapted cells to pH 4.5 (acid shock) and then activates a complex acid tolerance response (ATR) that increases the potential of* Salmonella* survival under extremely acid environments (pH 3.0 to 4.0) [144]. The ATR mechanism requires acid shock proteins including RpoS sigma factor and PhoPQ. It has been shown that RpoS and PhoPQ provide protection against inorganic acids, while regulators RpoS, iron regulatory protein Fur, and adaptive response protein Ada provide a major tolerance to stress of organic acids [142, 145, 146]. The PhoP locus is a crucial virulence determinant and* Salmonella pho*P strains are very sensitive to microbial peptides. Several genes, including* rpo*S, and some acid shock proteins and heat shock proteins are implicated in* Salmonella* virulence. Commonly isolated from chicken carcasses* S.* Kentucky shows more acid sensitivity (pH 5.5) than other* Salmonella* serovars (Enteritidis, Mbandaka, and Typhimurium) [107]. When virulence gene presence was surveyed, acid adaptive stress genes including* rpo*S,* fur*, and* pho*PQ were detected in* S.* Kentucky [107]. Virulent* S.* Typhimurium strains with mutations in the* rpoS* gene were unable to develop a full ATR and had significantly reduced virulence potential [147–149].

It is known that virulence can be activated by acetic acid stress through the* hil*A gene. Virulence gene expression using* hil*A in response to pH showed upregulation in strains Typhimurium 23595, Typhimurium 14028, Seftenberg, Heidelberg, Mbandanka, Montevideo, and Infantis [108, 150].

### 4.4. Desiccation

*Salmonella* is heat tolerant and persistent in nature and survives long periods of time in dry products but requires *a*_*w*_ > 0.93 for growth. Increasing numbers of multistate* Salmonella* outbreaks associated with dry foods have occurred [151, 152]. Some of these outbreaks have been characterized by a low infectious dose. It is believed that* Salmonella* has increased virulence potential induced by survival of other stresses including acid and heat. Salmonellae can be exposed to desiccation stress in the poultry farm environment by numerous factors. Persistence of* Salmonella* cells in poultry house surroundings, environment dust, dry fecal matter, and floor materials and equipment remaining contaminated after cleaning and sanitization procedures can expose* Salmonella* to desiccation. The incapacity to detect dormant* Salmonella* cells may undermine routine hygiene checks [153].

The genetic mechanism of* Salmonella* survival is related to the* proP* (Proline permease II) gene. When a* proP* deletion was assayed, mutants could not survive desiccation for long periods and became undetectable after four weeks. Sigma factor RpoS also plays a role in protecting cells from drying by stabilizing membranes and enzymes by trehalose synthesis, resulting in a more stable structure in the cell [151].

The formation of multicellular filamentous cells by* rdar* (red, dry, and rough colony) morphology is a major change induced in* Salmonella* by low *a*_*w*_ exposure.* Rdar* morphology promotes formation of aggregative fimbriae and cellulose increases desiccation resistance in* Salmonella* cells, and these can remain viable for months [154, 155]. The *a*_*w*_ of food matrices, product formulation, and storage temperature critically affect the survival of* Salmonella* in dry food matrices [156]. When bacteria are exposed to desiccation stress, the *a*_*w*_ in the cell is lowered. Strains Enteritidis, Typhimurium, and Mbandaka have been found to have greater persistence (over one year) than Seftenberg, but most authors agree that* S.* Seftenberg is the most tolerant to desiccation, surviving exposure to detergents and disinfectants up to 30 months [157–159].

More recently a cell shrinkage strategy for* Salmonella *has been studied as a mechanism of protection during desiccation. A scatter plot analysis showed that the conversion from rod shape to cocci occurred at a greater extent in* S.* Tennessee (strong desiccation resistance) than* S.* Typhimurium LT2 (weak desiccation resistance) responding to a 5-day desiccation treatment. Gene expression profile for the two serovars significantly differed with* S.* Tennessee having no change in genes involved in cell elongation (rodA, rodZ, mrdB, mreB, mrdA, mrcA, and mrcB) after 24 hours of desiccation while* S.* Typhimurium LT2 cell morphology genes were upregulated from 38- to 91-fold [160].

### 4.5. Fatty Acid Associated Genes

Adaptive mechanisms of* Salmonella* related to survival and virulence in low *a*_*w*_ foods include a modification of the fatty acid profile.* Salmonella* will induce and express genes encoding enzymes involved in the modification of the fatty acids, which will increase osmotolerance.

Increase in cyclopropane fatty acids is considered to be an indicator of starvation or desiccation stress [161]. Fatty acid profiles affect the lipid membrane and increase osmotolerance.* Salmonella enterica* increases membrane fluidity via* fab*A,* fab*B, and cfa pathway [162, 163]. Upregulation of short chain fatty acid related genes including* fabA*,* fabB*, and* cfa* was determined when* Salmonella* was inoculated in poultry feed [109]. Upregulation of fatty acid catabolic genes has been identified when* Salmonella* is exposed to dehydration stress under aerobic conditions [151, 164].

### 4.6. Cross Protection Effects

It is believed that cross protection between different factors including heat and acid stress can affect the virulence of* Salmonella*, although it is generally acknowledged that several genes, including* rpo*S, and some acid and heat shock proteins have related effects [148, 165]. For example, desiccation tolerance of* Salmonella enterica* can have a cross-tolerance effect for other stresses.* S.* Enteritidis,* S.* Newport,* S.* Infantis, and* S.* Typhimurium can show resistance to commonly used disinfectants, dry heat, and UV irradiation when exposed to a previous dehydration stress. The interaction between temperature and pH is also important. As cross protection effects can impact the survival and virulence of* Salmonella*, it is important to evaluate these factors during formulation, processing, and preservation of food products.

## 5. Conclusions

*Salmonella* serovars are resilient microorganisms with a complex genomic system that makes the organism able to react to different harsh environmental conditions at the farm, during processing and in the gastrointestinal tract. Different stress factors that the bacteria may be exposed to include temperature, pH, osmotic shifts, and low *a*_*w*_ beyond their normal growth range. More research is needed to understand why a few* Salmonella* serovars are responsible for a majority of human diseases and demonstrate such unique reservoirs and pathogenesis. With a better understanding of serovar specifity, mitigation methods can be implemented to control* Salmonella* at preharvest and postharvest levels.

## Figures and Tables

**Table 1:** Examples of some genomic characteristics of *Salmonella* serovars.

Serovar	Genome size (kb)	G + C (%)	Plasmid size (kb)		Reference
Abortusovis	4508	52.1			[15]
^ a^Agona	4762	52.1			[16]
Bovismorbificans	4896	52.1	2 plasmids, no sizes reported		[17]
Choleraesuis SC-1367	4755	52.11	pSC: 138	pSCV: 50	[18]
Cubana	4730	52.2	122	166	[19]
Durban	4678	52.2	59		[20]
^ a^Enteritidis PT4	4685	52.17			[21]
^ a^Gallinarum	4658	52.22			[21]
^ a^Manhattan	4684	52.17			[22]
^ a^Namur	4842	51.96			[23]
^ a^Oranienburg	4609	52.2			[24]
^ a^Paratyphi A (ATCC 9150)	4585	53			[25]
^ a^St. Paul	4624	52.1			[26]
^ a^Thompson	4707	52.2			[27]
Typhi CT18	4809	52.09	pHCM1: 218	pHCM2: 106	[28]
Typhimurium LT2	4857	53	94		[29]
^ a^Typhi Ty2	4792	52.02			[30]

^a^No plasmid was present in these strains.

**Table 2:** Number of *Salmonella* foodborne outbreaks in the US linked to animals from 2006 to 2011 [14].

Food animals	Number of outbreaks	Number of Illness
Poultry	145	2580
Eggs	117	2938
Pork	43	1043
Beef	37	1138
Dairy	21	682
Wild game	4	48

**Table 3:** Number of *Salmonella* foodborne outbreaks in the US linked to crops from 2006 to 2011 [14].

Food	Number of outbreaks	Number of Illnesses
Fruits/nuts	36	2359
Sprouts	21	711
Vine stalk vegetables	21	3216
Leafy vegetables	11	306
Roots	6	172
Grains/beans	5	259
Oil/sugar	1	14
Fungus	1	10

**Table 4:** Examples of *Salmonella* serovars isolated from foodborne outbreaks in the US in humans and most common food items related to each serovar from 2007 to 2011 [14].

Serovar	Number of outbreaks	%	Ill	Hospitalized	Deaths	Most common food vehicles
Enteritidis	167	27%	4972	394	2	Egg, chicken, pork, and beef
Typhimurium	84	14%	2043	342	9	Chicken, leafy greens, and peanut butter
Heidelberg	44	7%	1875	212	5	Chicken, turkey, and dairy products
Newport	63	10%	1581	209	2	Sprouts, vegetables, tomatoes, pork, and poultry
Montevideo	21	3%	1154	141	0	Beef, pepper, pork, and cheese
Braenderup	19	3%	203	29	1	Pork, chicken, and vegetables
Muenchen	17	3%	229	34	1	Sprouts, deli meat, and fruit
Infantis	16	3%	363	34	0	Pork, turkey, and beans
Javiana	14	2%	876	73	1	Chicken, pork, fruits, and vegetables
Saintpaul	10	2%	1866	340	2	Peppers, tomatoes, poultry, and beef

**Table 5:** Examples of *Salmonella* serovars (total % serotypes) profiles of *Salmonella* serovars isolated from broilers in the US [36].

*Salmonella* serovar	2000	2001	2002	2003	2004	2005	2006	2007	2008	2009
Kentucky	25.49	33.59	36.28	35.96	42.74	45.18	48.97	47.14	36.83	39.61
Enteritidis	2.68	1.62	3.13	3.51	6.06	7.71	13.66	10.82	18.31	20.78
Heidelberg	23.05	24.81	24.88	19.85	15.15	14.52	11.34	13.43	12.96	14.07
^ b^Typhimurium	6.4	6.39	4.37	6.05	5.22	9.45	8.08	8.96	11.52	6.49
^ a^I 4, 5, 12:i:-					3.03	4.18	4.3	2.49	3.29	2.16
Montevideo	4.31	3.05	1.9	2.06	2.09	3.47	1.63	2.24	2.06	1.73
Schwarzengrund	2.91	3.05	1.71		2.82	2.83	1.29		1.44	1.3
Typhimurium (var. Copenhagen)	6.64	3.34	6.36	9.56	8.78					
Hadar	4.89	2.96	4.37	1.82		1.03				
Thompson	3.14	2.48	2.18	2.06		1.16				
Infantis			1.33		1.25		1.03	1.49	2.06	

^a^Prior to 2004, isolates fitting the designation were included in the unidentified isolates category.

^b^After 2005 Typhimurium includes Typhimurium 5 (formerly Copenhagen).

**Table 6:** Examples of *Salmonella* serovars profiles from samples of ground chicken collected in the US [36].

*Salmonella* serovar	% total serotyped									
	2000	2001	2002	2003	2004	2005	2006	2007	2008	2009
Kentucky	26.53	18	16	20	12.89	31.91	42	24.81	28.57	30.88
Enteritidis	4.08		4.8		1.8	31.91	16	25.56	20	29.41
Heidelberg	18.37	26	29.6	25.71	1.55	12.77	16	20.3	24.76	10.29
Typhimurium	12.24	10	9.6	0.95	1.8	6.38	4	6.02	5.71	7.35
^ a^I 4, 5, 12:i:-					0.26	2.13	4	5.26	0.95	4.41
Braenderup					0.26					2.94
Infantis	4.08		3.2	3.81	0.52		3	2.26	1.9	1.47
Montevideo			4.8	1.9	1.29				1.9	
Schwarzengrund	2.04	20	3.2		1.29		3	1.5		
Hadar	6.12	4	3.2	27.62	0.26	2.13	1			
Thompson	4.08	4	3.2	5.71	1.03	2.13		2.26		

^a^Prior to 2004, isolates fitting the designation were included in the unidentified isolates category.

**Table 7:** Examples of characteristic features of enteric fever and nontyphoidal salmonellosis.

	Enteric fever	NTS
Natural host	Humans	Food animals, reptiles, and insects
Common related serovars	Typhi and Paratyphi	Enteritidis, Typhimurium, and Heidelberg
Incubation period	7–14 days	6–12 hours
Common symptoms	Fever, coated tongue, bradycardia, rose spots on chest, and myalgia	Nausea, vomiting, fever, chills, abdominal pain, and myalgia
Treatment	Fluoroquinone (5–7 days), chloramphenicol, and amoxicillin^a^	Antibiotic treatment not recommended for systemic disease; fluoroquinones^b^
Vaccination	Available in endemic areas^c^	Not available

^a^Depending on local patterns of antibiotic resistance, severity of the disease, availability, and cost.

^b^Fluroquinones are usually preferred if antibiotic treatment is appropriate.

^c^Licensed available vaccines. Efficacy of the vaccine is 60–80% and protection for up to 7 years.

**Table 8:** Examples of nontyphoidal *Salmonella* isolates from US patients and resistance profile of specific antimicrobial agents [75].

Serovar	Antimicrobial agent group													
	Cephems		Quinolones		Phenicols		Folate pathway inhibitors		Penicillins		Aminoglycosides		Tetracycline	
	Ceftriaxone		Naldixic Acid		Chloramphenicol		Sulfisoxazole		Ampicillin		Streptomycin		Tetracycline	
Newport	22	31%	1	2%	22	18%	23	10%	23	10%	25	12%	25	9%
Typhimurium	18	26%	5	10%	74	61%	105	47%	96	43%	94	44%	106	39%
Enteritidis			27	55%	3	2%	10	4%	12	5%	3	1%	11	4%
Heidelberg	15	21%			1	1%	7	3%	24	11%	17	8%	15	5%
Dublin	3	4%											22	8%
I 4, [5], 12:i:-	2	3%	4.1	8%	1	1%	15	7%	17	8%	15	7%		
Montevideo											2	1%	3	1%
Cubana	1	1%	1	2%										
Kentucky	1	1%	1	2%										
Choleraesuis			1	2%										
Paratyphi B					8	7%	9	4%	9	4%	10	5%	10	4%
Other					11	9%	41		31	14%	42	20%	68	25%

**Table 9:** Examples of nontyphoidal *Salmonella* isolates from US patients and their multidrug resistance profile [75].

Serovar	Multidrug							
	Resistant to >5 antimicrobials		ACSSuT^1^		ACSSuTAuCx^2^		ACT/S^3^	
Newport	22	17.2%	22	20.6%	22	66.7%	4	36.4%
Typhimurium	76	59.4%	68	63.6%	7	21.2%	4	36.4%
Heidelberg	6	4.7%	1	0.9%				
Dublin	3	2.3%	3	2.8%	3	9.1%	1	9.1%
I 4, [5], 12:i:-	3	2.3%	1	0.9%				
Infantis	1	0.8%	1	0.9%	1	3.0%		
Cubana	2	1.6%	1	0.9%			1	9.1%
Concord	2	1.6%						
Denver	1	0.8%						
Kentucky	2	1.6%						
Choleraesuis	2	1.6%	1	0.9%			1	9.1%
Paratyphi B	7	5.5%	7	6.5%				
Unknown	1	0.8%	1	0.9%				

^1^ACSSuT: ampicillin, chloramphenicol, streptomycin, sulfamethoxazole/sulfisoxazole, and tetracycline.

^2^ACSSuTAuCx: ACSSuT, amoxicillin-clavulanic acid, and ceftriaxone.

^3^ACT/S: ampicillin, chloramphenicol, and trimethoprim-sulfamethoxazole.

**Table 10:** Examples of *Salmonella* serovars profiles from cows and bulls in the US [36].

Serovar	% total serotyped									
	2000	2001	2002	2003	2004	2005	2006	2007	2008	2009
Montevideo	10	13.46	5.48	2.63	4.17	11.5	15.79	9.52	16.67	25
Newport	15	5.77	24.66	13.16	8.33	3.85		16.67	8.33	16.67
Agona			6.85	5.26	4.17	7.69	10.53		16.67	8.33
Kentucky	7.5	9.62	6.85			7.69	21.05	2.38	8.33	8.33
Mbandaka	2.5	3.85	4.11				5.26	2.38		8.33
Cerro				7.89	8.33	7.69	5.26	11.9	16.67	
Anatum		9.62		2.63	4.17	7.69		16.67	8.33	
Muenster	12		10.96	18.42	8.33	7.69	10.53	9.52	8.33	
Typhimurium	10	7.69	6.85	7.89	8.33	11.54				
Dublin	2.5	5.77			8.33	3.85	5.26			
Meleagridis		3.85		5.26	4.17	3.85	5.26	2.38		
Infantis	2.5		5.48	2.63	4.17	7.69		4.76		
Derby	2.5		4.11	5.26	8.33	3.85				
Enteritidis							5.26	2.38		

**Table 11:** Examples of *Salmonella* serovars profiles from steers and heifers in the US [36].

Serovars	% total serotyped									
	2000	2001	2002	2003	2004	2005	2006	2007	2008	2009
Dublin		18.18			8.33	16.67		22.22	22.22	
Montevideo	50	9.09	7.14	10.53			10	11.11	11.11	10
Typhimurium	25				8.33		10		11.11	10
Anatum				10.53	8.33		10	11.11	11.11	
Newport				5.26	8.33	8.32	20	11.11	11.11	
Mbandanka				5.26					11.11	
Muenster			7.14			8.32	10			10
Muenchen						16.67				10
Poona						16.67				10
Derby		36.36	7.14	15.79	33.33					
Heidelberg		9.09	7.14	5.26						
Kentucky		9.09	14.29	10.53				11.11		

**Table 12:** Examples of *Salmonella* serovars profiles from ground beef collected in the US [36].

Serovars	% total serotyped									
	2000	2001	2002	2003	2004	2005	2006	2007	2008	2009
Montevideo	12.72	14.05	11.32	10	14.06	13.89	16.86	23.43	24.51	31.1
Dublin				5.31	4.95	4.17	5.14	9.81	12.25	12.8
Newport	8.25	10.91	10.69	11.02	7.52	6.48	6.86	5.99	7.35	9.15
^ a^Typhimurium	6.31	5.53	4.07	5.51	4.16	9.26	6	5.18	6.62	8.54
Anatum	6.8	9.27	9.8	9.18	10.89	9.26	7.71	3.81	7.6	4.88
Cerro	5.05	3.89	3.82			3.7	6.29	4.9	5.15	4.88
Kentucky	4.27	6.88	4.83	4.69	4.16			2.72	4.41	4.88
Typhimurium (var. Copenhagen)	7.77	3.74	6.49	5.51	3.56					
Muenster	4.47	7.77	8.27	4.9	9.31	7.87	9.71	7.63	3.92	
Mbandaka	4.37	5.38	4.58	4.49	3.37	5.56	4	6.27	4.17	
Agona			6.62	5.92	7.13	3.24		4.09		

^a^After 2005 Typhimurium includes Typhimurium 5 (formerly Copenhagen).

**Table 13:** Examples of *Salmonella* serovars profiles from market hogs in the US [36].

Serovars	% total serotyped									
	2000	2001	2002	2003	2004	2005	2006	2007	2008	2009
Derby	22.6	33.01	30.38	17.22	28.34	29.8	18.49	13.3	21.1	19.44
^ a^Typhimurium	3.08	2.94	2.95	3.97		13.47	8.22	20.69	10.09	16.67
Johannesburg	8.22	3.59	2.95	4.64	3.64	3.67	9.59	9.85	4.59	9.26
Infantis	6.85	8.5	5.91	7.28	7.69	8.98	5.48	8.37	12.84	7.41
Anatum	3.42	7.19	5.49	5.3	10.93	5.31	21.58	6.4	5.5	5.56
Adelaide					4.05	3.27		4.93		4.63
Agona							3.42	3.94	5.5	4.63
Heidelberg	5.82	4.25	2.95	6.62		2.45	4.45			3.7
Saintpaul	2.4	4.58	5.91	5.3		4.49	5.48	6.4	6.42	3.7
Typhimurium (var. Copenhagen)	16.1	6.86	13.08	10.6	17					
Reading	2.4	4.25	3.38	3.31	3.24	4.08				

^a^After 2005 Typhimurium includes Typhimurium 5 (formerly Copenhagen).

## References

[1] Grimont P. A., Weill F. X. (2007). *Antigenic Formulae of the Salmonella Serovars*.

[2] Malorny B., Hauser E., Dieckmann R., Porwollik S. (2011). New approaches in subspecies-level *Salmonella* classification. *Salmonella From Genome to Function*.

[3] Tindall B. J., Grimont P. A. D., Garrity G. M., Euzéby J. P. (2005). Nomenclature and taxonomy of the genus *Salmonella*. *International Journal of Systematic and Evolutionary Microbiology*.

[4] Brenner F. W., Villar R. G., Angulo F. J., Tauxe R., Swaminathan B. (2000). *Salmonella* nomenclature. *Journal of Clinical Microbiology*.

[5] Jensen A. N., Hoorfar J. (2000). Immediate differentiation of *Salmonella*-resembling colonies on brilliant green agar. *Journal of Rapid Methods and Automation in Microbiology*.

[6] Abulreesh H. H., Annous B., Gurtler J. B. (2012). Salmonellae in the environment. *Salmonella—Distribution, Adaptation, Control Measures and Molecular Technologies*.

[7] Le Minor L. (1992). The genus *Salmonella*. *The Prokaryotes: A Handbook on the Biology of Bacteria: Ecophysiology, Isolation, Identification, Applications*.

[8] Sanchez S., Hofacre C. L., Lee M. D., Maurer J. J., Doyle M. P. (2002). Animal sources of salmonellosis in humans. *Journal of the American Veterinary Medical Association*.

[9] Rodriguez A., Pangloli P., Richards H. A., Mount J. R., Draughon F. A. (2006). Prevalence of *Salmonella* in diverse environmental farm samples. *Journal of Food Protection*.

[10] Hoelzer K., Switt A. I. M., Wiedmann M. (2011). Animal contact as a source of human non-typhoidal salmonellosis. *Veterinary Research*.

[11] Sanderson K. E., Demerec M. (1965). The linkage map of *Salmonella typhimurium*. *Genetics*.

[12] Batz M. B., Hoffmann S., Morris J. G. (2012). Ranking the disease burden of 14 pathogens in food sources in the United States using attribution data from outbreak investigations and expert elicitation. *Journal of Food Protection*.

[13] USDA Foodborne illness cost calculator: *Salmonella*. http://www.ers.usda.gov/topics/food-safety/foodborne-illness/salm_intro.asp.

[14] Center for Disease Control and Prevention (CDC) (2013). *Foodborne Outbreak Online Database (FOOD)*.

[15] Deligios M., Bacciu D., Deriu E. (2014). Draft genome sequence of the host-restricted *Salmonella enterica* serovar abortusovis strain SS44. *Genome Announcements*.

[16] McCusker M. P., Hokamp K., Buckley J. F., Wall P. G., Martins M., Fanning S. (2014). Complete genome sequence of *Salmonella enterica* serovar Agona pulsed-field type SAGOXB.0066, cause of a 2008 Pan-European outbreak. *Genome Announcements*.

[17] Gopinath G., Beaubrun J. J.-G., Grim C. (2014). Whole-genome sequences of six *Salmonella enterica* Serovar Bovismorbificans isolates associated with a 2011 multistate hummus-borne outbreak. *Genome Announcements*.

[18] Chiu C. H., Tang P., Chu C. (2005). The genome sequence of *Salmonella enterica* serovar Choleraesuis, a highly invasive and resistant zoonotic pathogen. *Nucleic Acids Research*.

[19] Hoffmann M., Muruvanda T., Pirone C. (2014). First fully closed genome sequence of *Salmonella enterica* subsp. *enterica* serovar cubana associated with a food-borne outbreak. *Genome Announcements*.

[20] Russell D. A., Bowman C. A., Hatfull G. F. (2014). Genome sequence of *Salmonella enterica* subsp. *enterica* Strain Durban. *Genome Announcements*.

[21] Thomson N. R., Clayton D. J., Windhorst D. (2008). Comparative genome analysis of *Salmonella enteritidis* PT4 and *Salmonella gallinarum* 287/91 provides insights into evolutionary and host adaptation pathways. *Genome Research*.

[22] Sassera D., Gaiarsa S., Scaltriti E. (2013). Draft genome sequence of *Salmonella enterica* subsp. *enterica* serovar Manhattan strain 111113, from an outbreak of human infections in Northern Italy. *Genome Announcements*.

[23] Barbau-Piednoir E., Bertrand S., Roosens N. H., de Keersmaecker S. C. J. (2014). Genome sequence of the *Salmonella enterica* subsp. *enterica* serovar namur strain 05-2929, lacking the *Salmonella* atypical fimbrial operon. *Genome Announcements*.

[24] Estrada-Acosta M., Medrano-Félix A., Jiménez M. (2013). Draft genome sequence of *Salmonella enterica* subsp. *enterica* serotype saintpaul strain S-70, isolated from an aquatic environment. *Genome Announcements*.

[25] McClelland M., Sanderson K. E., Clifton S. W. (2004). Comparison of genome degradation in Paratyphi A and Typhi, human-restricted serovars of *Salmonella enterica* that cause typhoid. *Nature Genetics*.

[26] Medrano-Félix A., Estrada-Acosta M., Jimenez M. (2013). Draft genome sequence of *Salmonella enterica* subsp. enterica serotype Oranienburg Strain S-76, isolated from an aquatic environment. *Genome Announcements*.

[27] Parker C. T., Gorski L., Huynh S. (2013). Complete genome sequence of *Salmonella enterica* subsp. *enterica* Serovar Thompson Strain RM6836. *Genome Announcements*.

[28] Parkhill J., Dougan G., James K. D. (2001). Complete genome sequence of a multiple drug resistant *Salmonella enterica* serovar Typhi CT18. *Nature*.

[29] McClelland M., Sanderson K. E., Spieth J. (2001). Complete genome sequence of *Salmonella enterica* serovar Typhimurium LT2. *Nature*.

[30] Deng W., Liou S.-R., Plunkett G. (2003). Comparative genomics of *Salmonella enterica* serovar Typhi strains Ty2 and CT18. *Journal of Bacteriology*.

[31] Alali W. Q., Thakur S., Berghaus R. D., Martin M. P., Gebreyes W. A. (2010). Prevalence and distribution of *Salmonella* in organic and conventional broiler poultry farms. *Foodborne Pathogens and Disease*.

[32] Bailey J. S., Stern N. J., Fedorka-Cray P. (2001). Sources and movement of *Salmonella* through integrated poultry operations: a multistate epidemiological investigation. *Journal of Food Protection*.

[33] Maciorowski K. G., Jones F. T., Pillai S. D., Ricke S. C. (2004). Incidence, sources, and control of foodborne *Salmonella* spp. in poultry feeds. *World's Poultry Science Journal*.

[34] Liljebjelke K. A., Hofacre C. L., Liu T. (2005). Vertical and horizontal transmission of *Salmonella* within integrated broiler production system. *Foodborne Pathogens and Disease*.

[35] Roy P., Dhillon A. S., Lauerman L. H., Schaberg D. M., Bandli D., Johnson S. (2002). Results of *Salmonella* isolation from poultry products, poultry, poultry environment, and other characteristics. *Avian Diseases*.

[36] Haley C. A., Dargatz D. A., Bush E. J., Erdman M. M., Fedorka-Cray P. J. (2012). *Salmonella* prevalence and antimicrobial susceptibility from the national animal health monitoring system swine 2000 and 2006 studies. *Journal of Food Protection*.

[37] Hennessy T. W., Hedberg C. W., Slutsker L. (1996). A national outbreak of *Salmonella enteritidis* infections from ice cream. *The New England Journal of Medicine*.

[38] Braden C. R. (2006). *Salmonella enterica* serotype enteritidis and eggs: a national epidemic in the United States. *Clinical Infectious Diseases*.

[39] Martelli F., Davies R. H. (2012). *Salmonella* serovars isolated from table eggs: an overview. *Food Research International*.

[40] Jones D. R., Musgrove M. T. (2007). Pathogen prevalence and microbial levels associated with restricted shell eggs. *Journal of Food Protection*.

[41] Howard Z. R., O'Bryan C. A., Crandall P. G., Ricke S. C. (2012). *Salmonella* Enteritidis in shell eggs:current issues and prospects for control. *Food Research International*.

[42] Rettger L. F. (1909). Further studies on fatal septicemia in young chickens, or ‘white diarrhea’. *The Journal of Medical Research*.

[43] Bullis K. L. (1977). The history of avian medicine in the U.S. II. Pullorum disease and fowl typhoid. *Avian Diseases*.

[44] Lister S. A., Barrow P., Pattison M. (2008). Bacterial diseases. *Poultry Diseases*.

[45] Hafez H. M., Jodas S. (2000). *Salmonella* infections in turkeys. *Salmonella in Domestic Animals*.

[46] Shivaprasad H. L., Methner U., Barrow P. A., Methner U., Barrow P. A. (2013). *Salmonella* infections in the domestic fowl. *Salmonella in Domestic Animals*.

[47] Shivaprasad H. L. (2000). Fowl typhoid and pullorum disease. *Revue Scientifique et Technique de l'OIE*.

[48] Boyd W. (2001). Making meat: science, technology, and American poultry production. *Technology and Culture*.

[49] Kabir S. M. L. (2010). Avian colibacillosis and salmonellosis: a closer look at epidemiology, pathogenesis, diagnosis, control and public health concerns. *International Journal of Environmental Research and Public Health*.

[50] Bäumler A. J., Hargis B. M., Tsolis R. M. (2000). Tracing the origins of *Salmonella* outbreaks. *Science*.

[51] Cogan T. A., Humphrey T. J. (2013). The rise and fall of *Salmonella* enteritidis in the UK. *Journal of Applied Microbiology*.

[52] Kumar Y., Sharma A., Sehgal R., Kumar S. (2009). Distribution trends of *Salmonella* serovars in India (2001–2005). *Transactions of the Royal Society of Tropical Medicine and Hygiene*.

[53] Rabsch W., Hargis B. M., Tsolis R. M. (2000). Competitive exclusion of *Salmonella enteritidis* by *Salmonella gallinarum* in poultry. *Emerging Infectious Diseases*.

[54] Connor B. A., Schwartz E. (2005). Typhoid and paratyphoid fever in travellers. *The Lancet Infectious Diseases*.

[55] Crump J. A., Ram P. K., Gupta S. K., Miller M. A., Mintz E. D. (2008). Part I. Analysis of data gaps pertaining to *Salmonella enterica* serotype Typhi infections in low and medium human development index countries, 1984–2005. *Epidemiology and Infection*.

[56] Crump J. A., Luby S. P., Mintz E. D. (2004). The global burden of typhoid fever. *Bulletin of the World Health Organization*.

[57] Fangtham M., Wilde H. (2008). Emergence of *Salmonella paratyphi* A as a major cause of enteric fever: need for early detection, preventive measures, and effective vaccines. *Journal of Travel Medicine*.

[58] Rabsch W., Tschäpe H., Bäumler A. J. (2001). Non-typhoidal salmonellosis: emerging problems. *Microbes and Infection*.

[59] Scallan E., Hoekstra R. M., Angulo F. J. (2011). Foodborne illness acquired in the United States—major pathogens. *Emerging Infectious Diseases*.

[60] Herikstad H., Motarjemi Y., Tauxe R. V. (2002). *Salmonella* surveillance: a global survey of public health serotyping. *Epidemiology and Infection*.

[61] Ginocchio C. C., Rahn K., Clarke R. C., Galán J. E. (1997). Naturally occurring deletions in the centisome 63 pathogenicity island of environmental isolates of *Salmonella* spp. *Infection and Immunity*.

[62] Jones T. F., Ingram L. A., Cieslak P. R. (2008). Salmonellosis outcomes differ substantially by serotype. *The Journal of Infectious Diseases*.

[63] Swartz M. N. (2002). Human diseases caused by foodborne pathogens of animal origin. *Clinical Infectious Diseases*.

[64] Su L.-H., Chiu C.-H., Chu C., Ou J. T. (2004). Antimicrobial resistance in nontyphoid *Salmonella* serotypes: a global challenge. *Clinical Infectious Diseases*.

[65] Hur J., Jawale C., Lee J. H. (2012). Antimicrobial resistance of *Salmonella* isolated from food animals: a review. *Food Research International*.

[66] Rajashekara G., Haverly E., Halvorson D. A., Ferris K. E., Lauer D. C., Nagaraja K. V. (2000). Multidrug-resistant *Salmonella* typhimurium DT104 in poultry. *Journal of Food Protection*.

[67] Martin L. J., Fyfe M., Doré K. (2004). Increased burden of illness associated with antimicrobial-resistant *Salmonella enterica* serotype Typhimurium infections. *The Journal of Infectious Diseases*.

[68] Davis M. A., Hancock D. D., Besser T. E. (2002). Multiresistant clones of *Salmonella enterica*: the importance of dissemination. *The Journal of Laboratory and Clinical Medicine*.

[69] Mather A. E., Reid S. W. J., Maskell D. J. (2013). Distinguishable epidemics of multidrug-resistant *Salmonella* typhimurium DT104 in different hosts. *Science*.

[70] Butaye P., Michael G. B., Schwarz S., Barrett T. J., Brisabois A., White D. G. (2006). The clonal spread of multidrug-resistant non-typhi *Salmonella* serotypes. *Microbes and Infection*.

[71] Michael G. B., Butaye P., Cloeckaert A., Schwarz S. (2006). Genes and mutations conferring antimicrobial resistance in *Salmonella*: an update. *Microbes and Infection*.

[72] Alcaine S. D., Warnick L. D., Wiedmann M. (2007). Antimicrobial resistance in nontyphoidal *Salmonella*. *Journal of Food Protection*.

[73] Sefton A. M. (2002). Mechanisms of antimicrobial resistance. *Drugs*.

[74] Foley S. L., Lynne A. M. (2008). Food animal-associated *Salmonella* challenges: pathogenicity and antimicrobial resistance. *Journal of Animal Science*.

[75] National Antimicrobial Resistance Monitoring System (NARMS) Executive report. http://www.fda.gov/AnimalVeterinary/SafetyHealth/AntimicrobialResistance/NationalAntimicrobialResistanceMonitoringSystem/ucm312356.htm.

[76] Dunne E. F., Fey P. D., Kludt P. (2000). Emergence of domestically acquired ceftriaxone-resistant *Salmonella* infections associated with AmpC *β*-lactamase. *The Journal of the American Medical Association*.

[77] Gupta A., Fontana J., Crowe C. (2003). Emergence of multidrug-resistant *Salmonella enterica* serotype Newport infections resistant to expanded-spectrum cephalosporins in the United States. *The Journal of Infectious Diseases*.

[78] Zhao S., Qaiyumi S., Friedman S. (2003). Characterization of *Salmonella enterica* serotype newport isolated from humans and food animals. *Journal of Clinical Microbiology*.

[79] Glynn M. K., Bopp C., Dewitt W., Dabney P., Mokhtar M., Angulo F. J. (1998). Emergence of multidrug-resistant *Salmonella enterica* serotype typhimurium DT104 infections in the United States. *The New England Journal of Medicine*.

[80] Acheson D., Hohmann E. L. (2001). Nontyphoidal salmonellosis. *Clinical Infectious Diseases*.

[81] Han J., David D. E., Deck J. (2011). Comparison of *Salmonella enterica* serovar Heidelberg isolates from human patients with those from animal and food sources. *Journal of Clinical Microbiology*.

[82] Angulo F. J., Mølbak K. (2005). Human health consequences of antimicrobial drug-resistant *Salmonella* and other foodborne pathogens. *Clinical Infectious Diseases*.

[83] la Ragione R., Metcalfe H. J., Villarreal-Ramos B., Werling D., Barrow P. A., Methner U. (2013). *Salmonella* infections in cattle. *Salmonella in Domestic Animals*.

[84] Wray C., Todd N., McLaren I., Beedell Y., Rowe B. (1990). The epidemiology of *Salmonella* infection of calves: the role of dealers. *Epidemiology and Infection*.

[85] Kahrs R. F., Bentinck-Smith J., Bjorck G. R., Bruner D. W., King J. M., Lewis N. F. (1972). Epidemiologic investigation of an outbreak of fatal enteritis and abortion associated with dietary change and *Salmonella typhimurium* infection in a dairy herd. A case report. *The Cornell Veterinarian*.

[86] Fone D. L., Barker R. M. (1994). Associations between human and farm animal infections with *Salmonella typhimurium* DT104 in Herefordshire. *Communicable Disease Report. CDR Review*.

[87] Uzzau S., Brown D. J., Wallis T. (2000). Host adapted serotypes of *Salmonella enterica*. *Epidemiology and Infection*.

[88] Blau D. M., McCluskey B. J., Ladely S. R. (2005). *Salmonella* in dairy operations in the United States: prevalence and antimicrobial drug susceptibility. *Journal of Food Protection*.

[89] Davies R. H., McLaren I. M., Bedford S. (1999). Observations on the distribution of *Salmonella* in a pig abattoir. *Veterinary Record*.

[90] Foley S. L., Lynne A. M., Nayak R. (2008). *Salmonella* challenges: prevalence in swine and poultry and potential pathogenicity of such isolates. *Journal of Animal Science*.

[91] USDA/FSIS Serotypes Profile of *Salmonella* Isolates from Meat and Poultry Products January 1998 through December 2011. http://www.fsis.usda.gov/wps/portal/fsis/topics/data-collection-and-reports/microbiology/annual-serotyping-reports.

[92] Boyen F., Haesebrouck F., Maes D., van Immerseel F., Ducatelle R., Pasmans F. (2008). Non-typhoidal *Salmonella* infections in pigs: a closer look at epidemiology, pathogenesis and control. *Veterinary Microbiology*.

[93] Wales A., Davies R. H., Barrow P. A., Methner U., Methner U., Barrow P. A. (2013). Environmental aspects of *Salmonella*. *Salmonella in Domestic Animals*.

[94] van de Giessen A. W. (1994). Intervention strategies for *Salmonella enteritidis* in poultry flocks: a basic approach. *International Journal of Food Microbiology*.

[95] Kinde H., Read D. H., Chin R. P. (1996). *Salmonella enteritidis*, phage type 4 infection in a commercial layer flock in southern California: bacteriologic and epidemiologic findings. *Avian Diseases*.

[96] Wales A., Breslin M., Davies R. (2006). Semiquantitative assessment of the distribution of *Salmonella* in the environment of caged layer flocks. *Journal of Applied Microbiology*.

[97] Jones F. T., Axtell R. C., Rives D. V. (1991). A survey of *Salmonella* contamination in modern broiler production. *Journal of Food Protectection*.

[98] Carramiñana J. J., Yangüela J., Blanco D. (1997). *Salmonella* incidence and distribution of serotypes throughout processing in a Spanish poultry slaughterhouse. *Journal of Food Protection*.

[99] Bailey J. S., Cox N. A., Craven S. E., Cosby D. E. (2002). Serotype tracking of *Salmonella* through integrated broiler chicken operations. *Journal of Food Protection*.

[100] Doyle M. P., Erickson M. C. (2006). Reducing the carriage of foodborne pathogens in livestock and poultry. *Poultry Science*.

[101] Hanning I. B., Nutt J. D., Ricke S. C. (2009). Salmonellosis outbreaks in the united states due to fresh produce: sources and potential intervention measures. *Foodborne Pathogens and Disease*.

[102] Lehmacher A., Bockemuhl J., Aleksic S. (1995). Nationwide outbreak of human salmonellosis in Germany due to contaminated paprika and paprika-powdered potato chips. *Epidemiology and Infection*.

[103] Werber D., Dreesman J., Feil F. (2005). International outbreak of *Salmonella* Oranienburg due to German chocolate. *BMC Infectious Diseases*.

[104] Doyle M. P., Buchanan R. L. (2013). *Salmonella* species. *Food Microbiology: Fundamentals and Frontiers*.

[105] Brockmann S. O., Piechotowski I., Kimmig P. (2004). *Salmonella* in sesame seed products. *Journal of Food Protection*.

[106] Cooke F., Threlfall E., Wain J. (2007). Current trends in the spread and occurrences of human salmonellosis: molecular typing and emerging antibiotic resistance *Salmonella*. *Molecular Biology and Pathogenesis*.

[107] Joerger R. D., Sartori C. A., Kniel K. E. (2009). Comparison of genetic and physiological properties of *Salmonella enterica* isolates from chickens reveals one major difference between serovar Kentucky and other serovars: response to acid. *Foodborne Pathogens and Disease*.

[108] González-Gil F., Le Bolloch A., Pendleton S., Zhang N., Wallis A., Hanning I. (2012). Expression of *hilA* in response to mild acid stress in *Salmonella enterica* is serovar and strain dependent. *Journal of Food Science*.

[109] Andino A., Pendleton S., Zhang N., Chen W., Critzer F., Hanning I. (2014). Survival of *Salmonella enterica* in poultry feed is strain dependent. *Poultry Science*.

[110] Kazmierczak M. J., Wiedmann M., Boor K. J. (2005). Alternative sigma factors and their roles in bacterial virulence. *Microbiology and Molecular Biology Reviews*.

[111] Spector M. P., Kenyon W. J. (2012). Resistance and survival strategies of *Salmonella enterica* to environmental stresses. *Food Research International*.

[112] Brumell J. H., Rosenberger C. M., Gotto G. T., Marcus S. L., Finlay B. B. (2001). SifA permits survival and replication of *Salmonella typhimurium* in murine macrophages. *Cellular Microbiology*.

[113] Yang Y., Khoo W. J., Zheng Q., Chung H.-J., Yuk H.-G. (2014). Growth temperature alters *Salmonella enteritidis* heat/acid resistance, membrane lipid composition and stress/virulence related gene expression. *International Journal of Food Microbiology*.

[114] Scott V. N., Chun Y., Kuehm J. (2009). Control of *Salmonella* in low-moisture foods I: minimizing entry of *Salmonella* into a processing facility. *Food Protection Trends*.

[115] Goepfert J. M., Biggie R. A. (1968). Heat resistance of *Salmonella typhimurium* and *Salmonella senftenberg* 775W in milk chocolate. *Applied Microbiology*.

[116] Moats W. A., Dabbah R., Edwards V. M. (1971). Survival of *Salmonella anatum* heated in various media. *Applied Microbiology*.

[117] Gibson B. (1973). The effect of high sugar concentrations on the heat resistance of vegetative micro-organisms. *Journal of Applied Bacteriology*.

[118] Mattick K. L., Jørgensen F., Wang P. (2001). Effect of challenge temperature and solute type on heat tolerance of *Salmonella* serovars at low water activity. *Applied and Environmental Microbiology*.

[119] Murphy R. Y., Marks B. P., Johnson E. R., Johnson M. G. (1999). Inactivation of *Salmonella* and Listeria in ground chicken breast meat during thermal processing. *Journal of Food Protection*.

[120] Doyle M. E., Mazzotta A. S. (2000). Review of studies on the thermal resistance of Salmonellae. *Journal of Food Protection*.

[121] Chen Z., Diao J., Dharmasena M., Ionita C., Jiang X., Rieck J. (2013). Thermal inactivation of desiccation-adapted *Salmonella* spp. in aged chicken litter. *Applied and Environmental Microbiology*.

[122] Churi A., Chalova V. I., Zabala-Díaz I. B., Woodward C. L., Ricke S. C. (2010). Increased temperature influences *hilA* gene fusion expression in a *Salmonella typhimurium* poultry isolate. *Food Biotechnology*.

[123] Park S. H., Jarquin R., Hanning I., Almeida G., Ricke S. C. (2011). Detection of *Salmonella* spp. survival and virulence in poultry feed by targeting the *hilA* gene. *Journal of Applied Microbiology*.

[124] Mañas P., Pagán R., Alvarez I., Usón S. C. (2003). Survival of *Salmonella* senftenberg 775 W to current liquid whole egg pasteurization treatments. *Food Microbiology*.

[125] Shah D. B., Bradshaw J. G., Peeler J. T. (1991). Thermal resistance of egg associated epidemic strains of *Salmonella enteritidis*. *Journal of Food Science*.

[126] Jeffreys A. G., Hak K. M., Steffan R. J., Foster J. W., Bej A. K. (1998). Growth, survival and characterization of CspA in *Salmonella enteritidis* following cold shock. *Current Microbiology*.

[127] Craig J. E., Boyle D., Francis K. P., Gallagher M. P. (1998). Expression of the cold-shock gene cspB in *Salmonella typhimurium* occurs below a threshold temperature. *Microbiology*.

[128] Kim B. H., Bang I. S., Lee S. Y. (2001). Expression of cspH, encoding the cold shock protein in *Salmonella enterica* serovar Typhimurium UK-1. *Journal of Bacteriology*.

[129] Rhodes M. W., Kator H. (1988). Survival of *Escherichia coli* and *Salmonella* spp. in estuarine environments. *Applied and Environmental Microbiology*.

[130] Lambert P. A., Fraise A. P., Lambert P. A., Maillard J. Y. (2008). Mechanisms of action of biocides. Principles and practice of disinfection. *Preservation and Sterilization*.

[131] Wales A. D., Allen V. M., Davies R. H. (2010). Chemical treatment of animal feed and water for the control of *Salmonella*. *Foodborne Pathogens and Disease*.

[132] McDonnell G., Russell A. D. (1999). Antiseptics and disinfectants: activity, action, and resistance. *Clinical Microbiology Reviews*.

[133] Solano C., García B., Valle J. (2002). Genetic analysis of *Salmonella enteritidis* biofilm formation: critical role of cellulose. *Molecular Microbiology*.

[134] White A. P., Surette M. G. (2006). Comparative genetics of the *rdar* morphotype in *Salmonella*. *Journal of Bacteriology*.

[135] Byrd J. A., DeLoach J. R., Corrier D. E., Nisbet D. J., Stanker L. H. (1999). Evaluation of *Salmonella* serotype distributions from commercial broiler hatcheries and grower houses. *Avian Diseases*.

[136] do Amaral L. A. (2004). Drinking water as a risk factor to poultry health. *Revista Brasileira de Ciência Avícola*.

[137] Poppe C., Barnum D. A., Mitchell W. R. (1986). Effect of chlorination of drinking water on experimental *Salmonella* infection in poultry. *Avian Diseases*.

[138] Capita R. (2007). Variation in *Salmonella* resistance to poultry chemical decontaminants, based on serotype, phage type, and antibiotic resistance patterns. *Journal of Food Protection*.

[139] Chung K. C., Goepfert J. M. (1970). Growth of *Salmonella* at low pH. *Journal of Food Science*.

[140] Tamblyn K. C., Conner D. E. (1997). Bactericidal activity of organic acids against *Salmonella typhimurium* attached to broiler chicken skin. *Journal of Food Protection*.

[141] Zottola E. A., Sasahara K. C. (1994). Microbial biofilms in the food processing industry—should they be a concern?. *International Journal of Food Microbiology*.

[142] Rychlik I., Barrow P. A. (2005). *Salmonella* stress management and its relevance to behaviour during intestinal colonisation and infection. *FEMS Microbiology Reviews*.

[143] Ernst R. K., Guina T., Miller S. I. (1999). How intracellular bacteria survive: surface modifications that promote resistance to host innate immune responses. *The Journal of Infectious Diseases*.

[144] Álvarez-Ordóñez A., Prieto M., Bernardo A., Hill C., López M. (2012). The acid tolerance response of *Salmonella* spp.: an adaptive strategy to survive in stressful environments prevailing in foods and the host. *Food Research International*.

[145] Foster J. W., Hall H. K. (1990). Adaptive acidification tolerance response of *Salmonella typhimurium*. *Journal of Bacteriology*.

[146] Bearson B. L., Wilson L., Foster J. W. (1998). A low pH-inducible, PhoPQ-dependent acid tolerance response protects *Salmonella typhimurium* against inorganic acid stress. *Journal of Bacteriology*.

[147] Leyer G. J., Johnson E. A. (1993). Acid adaptation induces cross-protection against environmental stresses in *Salmonella typhimurium*. *Applied and Environmental Microbiology*.

[148] Foster J. W., Spector M. P. (1995). How *Salmonella* survive against the odds. *Annual Review of Microbiology*.

[149] Lee I. S., Lin J., Hall H. K., Bearson B., Foster J. W. (1995). The stationary-phase sigma factor *σ*S (RpoS) is required for a sustained acid tolerance response in virulent *Salmonella typhimurium*. *Molecular Microbiology*.

[150] Durant J. A., Corrier D. E., Stanker L. H., Ricke S. C. (2000). Expression of the *hilA Salmonella typhimurium* gene in a poultry *Salm, enteritidis* isolate in response to lactate and nutrients. *Journal of Applied Microbiology*.

[151] Li H., Bhaskara A., Megalis C., Tortorello M. L. (2012). Transcriptomic analysis of *Salmonella* desiccation resistance. *Foodborne Pathogens and Disease*.

[152] Podolak R., Enache E., Stone W., Black D. G., Elliott P. H. (2010). Sources and risk factors for contamination, survival, persistence, and heat resistance of *Salmonella* in low-moisture foods. *Journal of Food Protection*.

[153] Sarlin L. L., Barnhart E. T., Moore R. W., Corrier D. E., Stanker L. H., Hargis B. M. (1998). Comparison of enrichment methods for recovery and chick infectivity of chlorine-injured *Salmonella enteritidis*. *Journal of Food Protection*.

[154] White A. P., Gibson D. L., Kim W., Kay W. W., Surette M. G. (2006). Thin aggregative fimbriae and cellulose enhance long-term survival and persistence of *Salmonella*. *Journal of Bacteriology*.

[155] Finn S., Condell O., McClure P., Amézquita A., Fanning S. (2013). Mechanisms of survival, responses, and sources of *Salmonella* in low-moisture environments. *Frontiers in Microbiology*.

[156] Troller J. A. (1986). Water relations of foodborne bacterial pathogens—an updated review. *Journal of Food Protection*.

[157] Davies R. H., Wray C. (1996). Persistence of *Salmonella enteritidis* in poultry units and poultry food. *British Poultry Science*.

[158] Kumar A., Kumar S. (2003). Survival kinetics of *Salmonella enterica* serotype senftenberg (*S*. senftenberg) after heat and acid stress. *World Journal of Microbiology and Biotechnology*.

[159] Pedersen T. B., Olsen J. E., Bisgaard M. (2008). Persistence of *Salmonella* Senftenberg in poultry production environments and investigation of its resistance to desiccation. *Avian Pathology*.

[160] Megalis C. Cell shrinkage strategy for *Salmonella* during desiccation.

[161] Kieft T. L., Ringelberg D. B., White D. C. (1994). Changes in ester-linked phospholipid fatty acid profiles of subsurface bacteria during starvation and desiccation in a porous medium. *Applied and Environmental Microbiology*.

[162] Baysse C., O’Gara F., Ramos J.-L., Filloux A. (2007). Role of membrane structure during stress signaling and adaptation in *Pseudomonas*. *Pseudomonas: A Model System in Biology*.

[163] Kim B. H., Kim S., Kim H. G., Lee J., Lee I. S., Park Y. K. (2005). The formation of cyclopropane fatty acids in *Salmonella enterica* serovar Typhimurium. *Microbiology*.

[164] Finn S., Händler K., Condell O. (2013). ProP is required for the survival of desiccated *Salmonella enterica* serovar typhimurium cells on a stainless steel surface. *Applied and Environmental Microbiology*.

[165] Leyer G. J., Johnson E. A. (1992). Acid adaptation promotes survival of *Salmonella* spp. in cheese. *Applied and Environmental Microbiology*.

